# Characteristics and Risk Factors of Myocardial Injury after Traumatic Hemorrhagic Shock

**DOI:** 10.3390/jcm11164799

**Published:** 2022-08-17

**Authors:** Xiujuan Zhao, Fuzheng Guo, Chu Wang, Zhenzhou Wang, Panpan Chang, Haiyan Xue, Tianbing Wang, Fengxue Zhu

**Affiliations:** 1 Department of Critical Care Medicine, Peking University People’s Hospital, Beijing 100044, China; 2 Trauma Medicine Center, Peking University People’s Hospital, Beijing 100044, China; 3 Key Laboratory of Trauma and Neural Regeneration (Peking University), Ministry of Education, Beijing 100044, China; 4 National Center for Trauma Medicine of China, Beijing 100044, China

**Keywords:** trauma, hemorrhage shock, myocardial injury, risk factors, acute kidney injury

## Abstract

Myocardial injury increases major adverse cardiovascular events and mortality in patients with traumatic hemorrhagic shock, but its prevalence and risk factors remain unclear. This study aimed to assess the prevalence and risk factors of myocardial injury after traumatic hemorrhagic shock. This was an observational, retrospective cohort study of patients with traumatic hemorrhagic shock at a tertiary university hospital from November 2012 to July 2021. Patient characteristics and clinical variables were recorded in 314 patients. The outcome was the occurrence of myocardial injury after traumatic hemorrhagic shock. Risk factors for myocardial injury were identified using logistic regression. The incidence of myocardial injury after the traumatic hemorrhagic shock was 42.4%, and 95.5% of myocardial injuries occurred within the first three days after trauma. In the multivariate analysis, the independent risk factors for myocardial injury after traumatic hemorrhagic shock included heart rate of >100 beats/min (OR [odds ratio], 3.33; 95% confidence interval [CI], 1.56–7.09; *p* = 0.002), hemoglobin level of <70 g/L (OR, 3.50; 95% CI, 1.15–10.60; *p* = 0.027), prothrombin time of >15 s (OR, 2.39; 95% CI, 1.12–5.10; *p* = 0.024), acute kidney injury (OR, 2.75; 95% CI, 1.27–5.93; *p* = 0.01), and a higher APACHE II score (OR, 1.08; 95% CI, 1.01–1.15; *p* = 0.018). The area under the receiver operating characteristic curve for the prediction of myocardial injury after a traumatic hemorrhagic shock was 0.67 (95% CI, 0.68–0.79) for a heart rate of >100 beats/min, 0.67 (95% CI, 0.61–0.73) for hemoglobin level of <70 g/L, 0.66 (95% CI, 0.60–0.73) for prothrombin time of >15 s, 0.70 (95% CI, 0.64–0.76) for acute kidney injury, and 0.78 (95% CI, 0.73–0.83) for APACHE II scores. The incidence rate of myocardial injury in traumatic hemorrhagic shock is high, and heart rates of >100 beats/min, hemoglobin levels of <70 g/L, prothrombin times of >15 s, AKI and higher APACHE II scores are independent risk factors for myocardial injury after traumatic hemorrhagic shock. These findings may help clinicians to identify myocardial injury after traumatic hemorrhagic shock early and initiate appropriate treatment.

## 1. Introduction

In a study on the changes in the causes of death among Chinese residents (1990–2017), trauma ranked fifth among the top ten causes of death and is still the main death threat and disease burden for Chinese people [1]. There are three death peaks associated with post-traumatic death. The first and second death peaks are closely related to massive blood loss and severe brain injury, while the third peak is often associated with sepsis, septic shock, and multiple organ failure; however, few studies have classified and analyzed the cause of death or the initial sequence of organ dysfunction in detail [2]. At present, with the popularization of the concept of damage control resuscitation and the innovation in hemostasis technology, more patients survive from the first two death peaks and enter the third peak [2]. Cardiac dysfunction is increasingly common in the treatment process and has become an important factor in determining prognosis. A previous study showed that over 50% of the patients with severe trauma developed cardiovascular dysfunction within 48 h after admission, and 20% of the patients died [3]. Even after discharge, the risk of subsequent death in trauma patients is twice that in people of the same age and sex. Trauma maintains high mortality, especially within 10 years, and nearly 25% of the deaths are related to cardiovascular disease [4].

Chest injury is considered the direct cause of myocardial injury in trauma patients. However, an increasing number of studies have found no history of direct chest injury in patients with trauma-related myocardial injury [5]. Studies have also found that patients with pelvic or abdominal injuries have twice the risk for post-traumatic myocardial infarction [5]. In addition, an increasing amount of indirect and non-traumatic clinical evidence has shown that cardiac dysfunction is related to critical diseases [3]. The occurrence and development of myocardial injury have been confirmed in patients with postpartum hemorrhage, acute critical illness, and sepsis and are closely related to high mortality and poor long-term prognosis [3]. However, the pathophysiological mechanism underlying the occurrence and development of post-traumatic myocardial injury remains unclear. Further, animal studies have shown that shock and inflammatory cascade caused by trauma are the main causes of myocardial cell structure and function damage [6].

Clinical studies have proved the existence of trauma-related myocardial injury and its close relationship with poor prognosis through laboratory indicators, such as myocardial injury markers, cardiac troponin I (cTNI), and B-type natriuretic peptide (BNP) [3]. However, research on prevalence and risk factors of myocardial injury in patients with traumatic hemorrhagic shock are lacking. The purpose of this study was to investigate the clinical characteristics and risk factors for myocardial injury after traumatic hemorrhagic shock. Furthermore, this study will provide a basis for early identification and intervention of myocardial injury and assist clinicians in identifying patients for immediate treatment to prevent further deterioration of the disease, ultimately reducing mortality.

## 2. Materials and Methods

### 2.1. Study Design

This was an observational, retrospective cohort study of patients with traumatic hemorrhagic shock at a tertiary university hospital from November 2012 to July 2021. This study was approved by the Peking University People’s Hospital Medical Ethics Committee (approval number 2020PHB258-01), and this ethics committee agreed to waive the need for informed consent because no treatment interventions were mandated, and no protected health information was collected or analyzed. All data were anonymized prior to the analysis. This study is in accordance with the Strengthening the Reporting of Observational Studies in Epidemiology (STROBE) statement [7] and the Declaration of Helsinki.

### 2.2. Patients

Patients were enrolled if they were aged ≥18 years, had undergone traumatic hemorrhagic shock, and had an expected length of stay of >72 h. Traumatic hemorrhagic shock was defined as meeting the following items at admission: obvious bleeding at any part of the body caused by trauma, hemoglobin level of <100 g/L or >30 g/L lower than that before trauma, and systolic blood pressure of <90 mmHg (or shock index [heart rate/systolic blood pressure] of >1) for three consecutive times or serum lactate level of >2 mmol/L. The exclusion criteria were pregnancy, shock due to other causes (e.g., cardiogenic shock or obstructive shock), non-traumatic patients, patients expected to die within 72 h of admission due to fatal trauma (e.g., fatal traumatic brain injury), and patients with incomplete data.

### 2.3. Data Collection

Only human data were collected consecutively and retrospectively. All data were obtained from the Trauma Specific Database of the hospital, a real-world and clinical database from 2012 to July 2021, cataloging data on >17,000 trauma cases. Myocardial injury and clinical characteristics were documented. Data quality was checked by reviewing a random sample of 10% of the data.

### 2.4. Potential Confounders and Risk Factors

We collected data on patient characteristics and potential variables for myocardial injury due to traumatic hemorrhagic shock. These variables included demographic characteristics, comorbidities (diabetes mellitus, hypertension, coronary artery disease, cerebral hemorrhage, and gastrointestinal hemorrhage), previous medication (antiplatelet drugs, anticoagulant drugs, and nonsteroidal anti-inflammatory drugs), causes of trauma (falling from height, road traffic accident, falling from a standing position, and others), main bleeding site (thoracic, abdominal, pelvic, limbs, and others), worsened vital signs (mean arterial pressure and heart rate) within 72 h after trauma, and worsened values of laboratory data (hemoglobin levels, platelet counts, leukocyte counts, neutrophil counts, serum creatinine levels, serum total bilirubin levels, prothrombin time, fibrinogen levels, D-dimer levels, pO_2_/FiO_2_ ratio, serum lactate levels, serum chloride levels, uric acid levels, C-reactive protein, serum procalcitonin, and BNP) within 72 h after trauma. Left ventricular ejection fraction (LVEF) on echocardiography within 72 h after trauma was recorded. Organ dysfunction (acute kidney injury, acute liver injury, and acute respiratory distress syndrome), injury severity scores (ISS), and acute physiology and chronic health evaluation II (APACHE II) scores within 72 h after trauma were evaluated.

Acute respiratory distress syndrome was defined according to the Berlin definition [8]. Acute kidney injury was defined by a serum creatinine increase of ≥0.3 mg/dL (≥26.5 μmol/L) within 48 h or urine volume of <0.5 mL/(kg·h) for 6 h [9]. Acute liver injury was defined as a serum total bilirubin level of >34.2 μmol/L or a serum alanine aminotransferase and aspartate aminotransferase level more than twice as high as the normal value, a liver function test performed twice that resulted as abnormal after injury, and no history of acute and chronic hepatitis or liver cirrhosis [10]. If such organ dysfunction occurred, effective treatment was given according to corresponding guidelines or treatment norms [8,9,10].

### 2.5. Outcome

The primary outcome was the occurrence of myocardial injury after traumatic hemorrhagic shock. Myocardial injury after traumatic hemorrhagic shock was defined as myocardial injury caused by ischemia or hypoxia that may or may not result in necrosis after non-cardiac trauma and occurred after traumatic hemorrhagic shock. The definition of myocardial injury was cTNI concentrations above the 99th percentile upper reference limit (URL). The injury was considered acute if there was a rise and/or fall of cTNI values [11]. cTNI levels were assessed via high-sensitivity troponin I assay on a DxI800 (Beckman Coulter, Brea, CA, USA), wherein the 99th percentile for this test was 0.034 ng/mL. Myocardial injury mainly occurs within 7 days after traumatic hemorrhagic shock; therefore, we detected the cTNI level within 7 days after traumatic hemorrhagic shock. Myocardial infarction was defined as clinical evidence of acute myocardial ischemia with detection of a rise and/or fall of cTNI values with at least one value above the 99th percentile URL and at least one of the following: symptoms of myocardial ischemia; new ischemic electrocardiogram changes; development of pathological Q waves; imaging evidence of new loss of viable myocardium or new regional wall motion abnormality in a pattern consistent with an ischemic etiology; and/or identification of a coronary thrombus by angiography or autopsy (not for types 2 or 3 myocardial infarctions) [11]. For patients with myocardial injury after traumatic hemorrhagic shock, treatments such as control of bleeding, improvement of oxygenation and tissue perfusion, anticoagulation after control of bleeding, and cardiovascular drugs (e.g., antiplatelets, statins, beta-blockers, and angiotensin-converting enzyme inhibitors) were given according to guidelines for bleeding following major trauma and guidelines for myocardial infarction [12,13].

### 2.6. Statistical Analysis

The NCSS-PASS 11 sample size estimation software was used to calculate the sample size of the logistic regression model. This study mainly investigated the relationship between myocardial injury after traumatic hemorrhagic shock and acute kidney injury (AKI). The AKI rate was thought to be 20% among patients with myocardial injury after traumatic hemorrhagic shock. We expected a sample size large enough to detect an odds ratio (OR) of 2.5, with 85% power at the 0.1 significance level using a two-sided test. After the calculation, the required sample size was 275.

Continuous and categorical variables are expressed as mean ± standard deviation (SD), median (25th percentile, 75th percentile), and percentages. Comparison of two independent samples, a Student’s t-test, or a nonparametric Mann–Whitney U test were used for continuous variables, and a Pearson χ2 test was used for categorical variables. The rates of missing data were <5% for all variables, missing continuous variables were reckoned to the median non-missing value, and missing categorical variables were reckoned to the value of the most frequent group.

Collinearity diagnostics were assessed for all risk factors. If tolerance was <0.1, or the variance inflation factor was >10, high levels of collinearity were determined, and variables needed to collapse into a single variable, or one of the two variables must have been removed from the model. The remaining variables were included into a logistic regression model to identify independent risk factors for myocardial injury after traumatic hemorrhagic shock. A logistic regression model was built using a backward stepwise selection procedure in which the presence of a myocardial injury after traumatic hemorrhagic shock was a dependent variable. Selection of independent variables for the model was based on bivariate analysis (*p* < 0.05) and collinearity between the variables (tolerance < 0.1). The cutoff for variable removal was set at a significance level of 0.05. The adjusted ORs and corresponding 95% confidence intervals (CIs) were calculated. The Hosmer–Lemeshow goodness-of-fit statistic was used to assess the calibration of the logistic regression model. The C-statistic expressed as a percentage (area under the receiver operating characteristic curve (AUC)) was used to assess the discrimination of the model. Potential confounders were entered as covariates in the logistic regression model.

For all analyses, a two-sided *p* value of <0.05 was considered statistically significant. Statistical analysis was conducted using SPSS 25.0 for Windows (SPSS, Chicago, IL, USA).

## 3. Results

### 3.1. Studies Included

**Figure 1:**
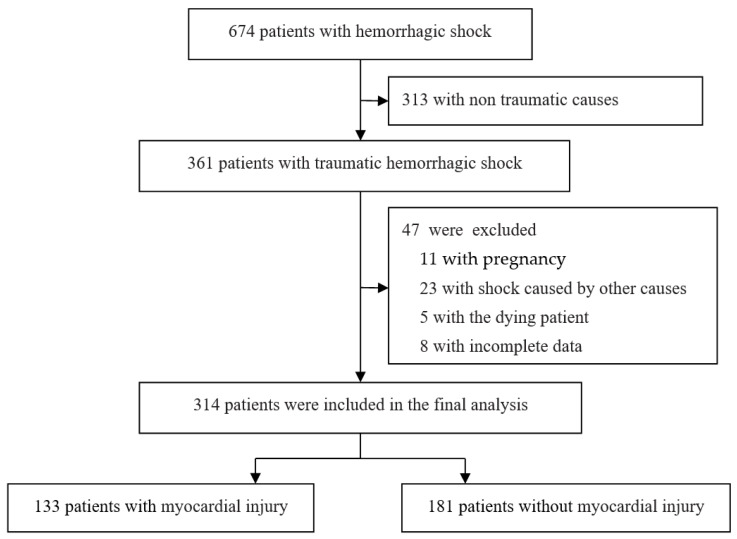
Patient flowchart.

**Figure 2:**
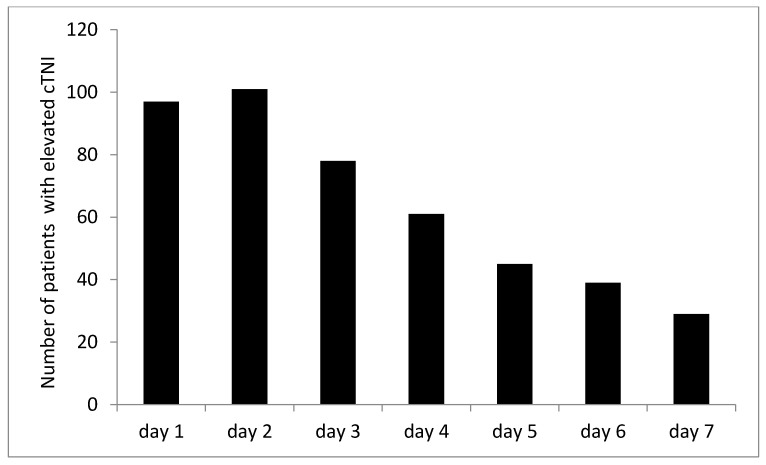
Number of patients with elevated cardiac troponin I in the first 7 days after trauma.

Among the 674 patients with hemorrhagic shock recruited in this hospital, 361 met the inclusion criteria. Finally, data from 314 patients were included in the analysis. The reasons for exclusion of the remaining patients are shown in Figure 1. The demographic and clinical indicators of the patients are presented in Table 1. In this study, myocardial injury after traumatic hemorrhagic shock occurred in 133 (42.4%) of 314 patients with traumatic hemorrhagic shock, mainly in the first 3 days (127/133, 95.5%) after trauma. The number of patients with elevated cTNI was the highest on the second day after trauma and then decreased gradually (Figure 2). Only two patients had myocardial infarction, and 88 patients (28.0%) developed AKI after traumatic hemorrhagic shock. According to the KDIGO staging criteria for AKI, stage 1 accounted for 81.8% (72/88), stage 2 accounted for 13.6% (12/88), and stage 3 accounted for 4.5% (4/88) of the 88 patients. The mortality rate of patients with traumatic hemorrhagic shock and myocardial injury was 23/133 (17.3%), while that of patients without myocardial injury was 7/181 (3.9%).

### 3.2. Risk Factors for Myocardial Injury after Traumatic Hemorrhagic Shock

Table 2 shows the comparison of risk factors between the myocardial injury group and non-myocardial injury group in patients with traumatic hemorrhagic shock. There were 23 risk factors associated with myocardial injury after traumatic hemorrhagic shock: male sex, falling from height, abdominal bleeding, bleeding of limbs, mean arterial pressure and heart rate 72 h after admission, laboratory data (leukocyte and platelet counts; prothrombin time; and hemoglobin, serum creatinine, fibrinogen, D-dimer, pO_2_/FiO_2_ ratio, serum lactate, serum chloride, uric acid levels, and serum procalcitonin levels) 72 h after admission, organ dysfunction (acute kidney injury, acute liver injury, and acute respiratory distress syndrome), and APACHE II scores and ISS within 72 h after admission.

**Figure 3:**
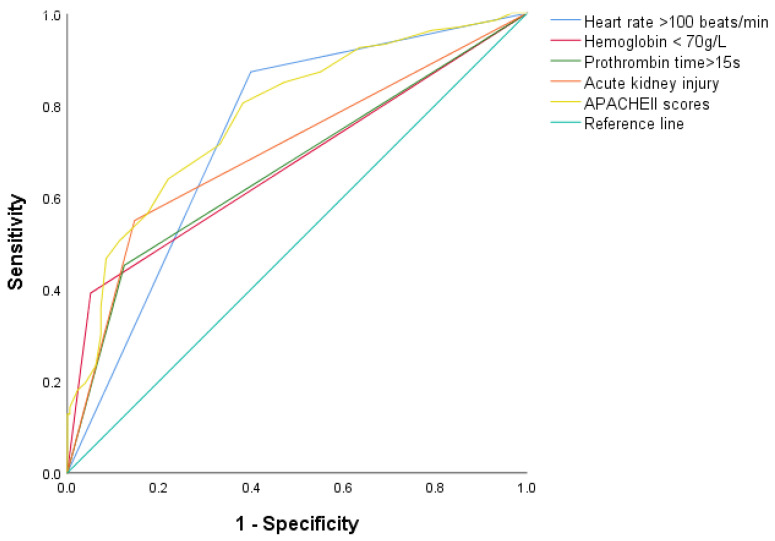
Receiver operating characteristic curve analysis of risk factors for the prediction of myocardial injury after traumatic hemorrhagic shock. The AUC for prediction of myocardial injury after traumatic hemorrhagic shock was 0.67 (95% CI, 0.68–0.79) for a heart rate of >100 beats/min, 0.67 (95% CI, 0.61–0.73) for a hemoglobin level of <70 g/L, 0.66 (95% CI, 0.60–0.73) for a prothrombin time of >15 s, 0.70 (95% CI, 0.64–0.76) for acute kidney injury, and 0.78 (95% CI, 0.73–0.83) for APACHE II scores.

According to collinearity diagnostics, no correlation was found. As a result, none of the variables was removed. All statistically significant variables were enrolled into a logistic regression model using myocardial injury after traumatic hemorrhagic shock as the dependent variable (Table 2). The independent risk factors for myocardial injury after traumatic hemorrhagic shock were a heart rate of >100 beats/min (OR, 3.33; 95% CI, 1.56–7.09; *p* = 0.002), hemoglobin levels of <70 g/L (OR, 3.50; 95% CI, 1.15–10.60; *p* = 0.027), prothrombin time of >15 s (OR, 2.39; 95% CI, 1.12–5.10; *p* = 0.024), AKI (OR, 2.75; 95% CI, 1.27–5.93; *p* = 0.01), and a higher APACHE II score (OR, 1.08; 95% CI, 1.01–1.15; *p* = 0.018) (Table 3). The AUC for the prediction of myocardial injury after traumatic hemorrhagic shock was 0.67 (95% CI, 0.68–0.79) for a heart rate of >100 beats/min, 0.67 (95% CI, 0.61–0.73) for hemoglobin levels of <70 g/L, 0.66 (95% CI, 0.60–0.73) for prothrombin time of >15 s, 0.70 (95% CI, 0.64–0.76) for AKI, and 0.78 (95% CI, 0.73–0.83) for APACHE II scores (Figure 3), and good calibration was shown in a regression model (Hosmer–Lemeshow χ^2^ = 3.798; *p* = 0.875).

### 3.3. Relevant Factors for Death in Patients with Myocardial Injury after Traumatic Hemorrhagic Shock

Among 133 patients with myocardial injury after traumatic hemorrhagic shock, 110 patients survived, and 23 patients died. Compared with patients who survived, those who died had lower mean arterial pressure (58.7 (51.0–67.3) vs. 67.3 (53.3–77.3) mmHg, *p* = 0.026), lower fibrinogen levels (145.0 (59.3–303.8) vs. 174.5 (128.0–297.8) mg/dL, *p* = 0.004), higher D-dimer levels (28,341.0 (6717.0–53,862.5) vs. 5870.0 (2425.0–14,384.0) ng/mL, *p* = 0.008), higher serum lactate levels (6.7 (2.7–11.3) vs. 3.0 (1.8–3.9) mmol/L, *p* = 0.003), higher cTNI levels (2.05 (0.99–3.34) vs. 0.11 (0.04–0.34) ng/mL, *p* < 0.001), a higher percentage with AKI (69.6% vs. 35.5%, *p* = 0.003), and higher APACHE II scores (27.0 (22.0–35.0) vs. 18.5 (15.0–22.0), *p* < 0.001). Due to the small sample size, it was not suitable for multivariate regression analysis to further confirm the relevant factors of death.

## 4. Discussion

We found that the incidence rate of myocardial injury in patients with traumatic hemorrhagic shock was 42.4%, and the in-hospital mortality rate of patients with myocardial injury was 17.3%, which was four times higher than that of patients without myocardial injury. Moreover, the heart rate at admission of >100 beats/min, hemoglobin levels of <70 g/L, prothrombin time of >15 s, occurrence of AKI, and higher APACHE II score were independent risk factors for myocardial injury.

A previous study reported that the incidence of trauma-related myocardial injury was 13.3% [3], which was significantly lower than the results of this study. This is because the population included in our study experienced shock, which led to a higher incidence. This phenomenon also occurred in a study of post-traumatic AKI. The incidence of AKI in patients with overall trauma was 13%, while that in the specific group with hemorrhagic shock caused by trauma could be as high as 42%, which was the latest incidence reported in the literature [14]. In this study, 88 (28%) of 314 patients with traumatic hemorrhagic shock had AKI, of which AKI stage I (kidney stage) accounted for the highest proportion (81.8%); moreover, 55 patients (62.5%) with AKI had myocardial injury. After multivariate analysis, it was first confirmed that AKI was an independent risk factor for myocardial injury in patients with traumatic hemorrhagic shock and had a high predictive value. In the study of perioperative myocardial injury, a stepwise correlation was found between glomerular filtration rate and the incidence of myocardial injury [15]. A lower glomerular filtration rate yielded a higher the risk of myocardial injury. It is known that when the body is in shock, the blood flow to non-important organs (such as skin, intestine, and kidney) is significantly reduced to ensure the blood supply to the heart and brain. The essence of this study is that insufficient tissue perfusion and the continuous accumulation of oxygen debt caused by shock lead to the occurrence of AKI. Rixen’s research confirms this view [16]. The essence of shock may partly explain the relationship between AKI and myocardial injury; however, toxin accumulation, liquid overload, electrolyte disorder, and acid–base imbalance after AKI may also play a key role in the process of myocardial injury [17]. Therefore, it is necessary to further study the relationship between AKI and myocardial injury. Since AKI was a risk factor for myocardial injury after traumatic hemorrhagic shock, timely measures such as hydration and infusion of blood products to improve perfusion, ensure oxygenation, and avoid nephrotoxic agents can be taken to prevent AKI and to reduce the occurrence of myocardial injury after traumatic hemorrhagic shock.

Although there are few studies on trauma-related myocardial injury, scholars began research on perioperative myocardial injury in non-cardiac surgery many years ago and formed a consensus [18]. Among the multiple studies on myocardial injury after non-cardiac surgery, the most famous is the VISION study (the vascular events in non-cardiac surgery patients cohort evaluation study), which included 15,133 patients, of which 1200 (8%) had myocardial injury [18]. At present, it is considered that the main cause of myocardial injury after non-cardiac surgery is the mismatch between oxygen supply and oxygen consumption. The overall incidence is approximately 8–18% according to the operation risk, and >90% of postoperative myocardial injury has no symptoms [19]. According to The Fourth Universal Definition of Myocardial Infarction, the occurrence of perioperative myocardial injury is classified as type 2 myocardial infarction [11]. Perioperative tachycardia, hypotension, bleeding, arrhythmia, and the increase in inflammatory cytokines, catecholamines, and cortisol caused by surgical trauma are risk factors for myocardial injury [15]. We believe that the occurrence of post-traumatic myocardial injury belongs to type 2 myocardial infarction, which is similar to the occurrence and development of postoperative myocardial injury. In this study, the median heart rate of patients with myocardial injury at admission was 116 beats/min, which was significantly higher than that of the group without myocardial injury. In the multivariate analysis, a heart rate of >100 beats/min was an independent risk factor for myocardial injury, which was consistent with the research results in VISION. Tachycardia was considered to reduce the duration of cardiac diastole, thus limiting the perfusion time of the coronary artery and increasing heart wall pressure and oxygen consumption, resulting in myocardial injury.

Similarly, at this level of oxygen supply, anemia caused by blood loss leads to a decline in oxygen-carrying capacity, and insufficient oxygen supply promotes the occurrence of myocardial injury. Some studies have noted that 30–40% of patients with acute myocardial infarction have significant anemia at admission [20]. In addition, the increase in free hemoglobin in plasma due to the fragmentation of red blood cells, the changed redox state of red blood cells during anemia, the decrease in free glutathione, and the decrease in nitric oxide can damage the diastolic function of blood vessels and the systolic function of ventricles, thus affecting myocardial function [21,22]. This study also found that the hemoglobin level in the myocardial injury group was significantly lower than that in the non-myocardial injury group. Moreover, it was confirmed that a hemoglobin level of <70 g/L was an independent risk factor for myocardial injury.

We believe that the pathophysiological mechanism of myocardial injury in people with traumatic shock is complex. In the case of myocardial injury in patients with coronavirus disease 2019, there are many mechanisms, such as cytokine storm, coagulation and immune disorders, and direct injury of endothelial cells and cardiomyocytes, in addition to the mechanism of myocardial oxygen supply imbalance [23]. De’Ath HD’s study confirmed the relationship between cardiac muscle injury and inflammation in trauma patients [24]. Their results found that the levels of TNF- α, IL-6, and IL-8 in patients with myocardial injury were significantly higher than those in patients without myocardial injury [24]. In addition, a study by Naganatar found that in trauma patients, high levels of epinephrine and norepinephrine in plasma at admission were closely related to the occurrence of myocardial injury [25]. Wall’s study investigated the pathophysiology of cardiac dysfunction after trauma by implementing a clinically relevant murine model of post-traumatic hemorrhage cardiac dysfunction [6]. At the histological level, a large number of neutrophils, macrophages, and monocytes were found in cardiomyocytes, and significant morphological changes occurred in the myocardium, including myocardial interstitial edema, widespread disorganization of the cardiac myofibrillar ultrastructure, and a relaxation of the sarcomere in the cardiomyocytes [6]. When the body suffers from infection or tissue damage, the coordinated activation of inflammation and coagulation is a systematic defense mechanism that can be traced back to early invertebrates [26]. There is sufficient evidence that injury and bleeding can activate inflammation, resulting in an increase in cytokines, stimulation and recruitment of immune cells, and increased tissue factor expression in endothelial cells and monocytes [27]. In animal experiments, a strong correlation has been demonstrated between prothrombin time, activated partial thromboplastin time, and IL-10 and IL-1 [28]. The survival rate of trauma patients can be improved by downregulating the expression of related cytokines and regulating the inflammation and coagulation response caused by trauma [28]. In trauma patients, trauma-induced coagulation often occurs in patients with the most severe injury and shock, suggesting that excessive activation of the sympathetic adrenal system under strong stress promotes the emergence of early coagulation disorders [29]. In this study, it was found that prothrombin time and D-dimer levels were significantly higher in the myocardial injury group than those in the non-myocardial injury group. In the myocardial injury group, ISS score, lactic acid, leukocyte count, and procalcitonin levels were also higher than those in the non-myocardial injury group, suggesting that the degree of trauma and shock was more severe, and the body stress response was more intense.

In recent years, some scholars have found a potential unifying pathological link between sympathoadrenal hyperactivation, endotheliopathy, and poor outcome in different types of acute critical illness (severe trauma, sepsis, myocardial infarction, or post-cardiac arrest syndrome) [30]. They described this proposed disease entity as shock-induced endotheliopathy [30]. They believe that the potential causal relationship between them is that the excessive activation of sympathetic adrenal function caused by shock leads to the injury of the glycocalyx in the vascular endothelium, resulting in a series of lesions, including increased vascular permeability, microvascular thrombosis, thromboinflammation, and organ failure due to reduced oxygen supply [30]. Some scholars have proposed the hypothesis that β-blocker therapy can prevent or reduce catecholamine-induced endothelial lesions, thereby improving the survival rate of patients with heart disease owing to various causes (trauma, sepsis, myocardial infarction) [30]. In this study, we found that the incidence of myocardial injury in traumatic hemorrhagic shock was as high as 42.4% and often occurred within 3 days after trauma, and the related mortality could be as high as 17.3%. We believe that in addition to shock, severe trauma-induced stress, systemic inflammatory response, and traumatic coagulopathy contributed to the high incidence rate of myocardial injury in this group. Therefore, trauma physicians should be vigilant when receiving patients, immediately identify and correct the state of shock, and avoid irreversible heart failure and kidney failure.

Based on previous studies, the incidence rate of myocardial injury was higher in patients with traumatic shock, especially in cases of chest, abdomen, and pelvic injury [5]. These injuries often lead to massive bleeding and early coagulation dysfunction, which manifests as heart rate increase, hemoglobin reduction, blood pressure reduction, oliguria or no urine and acidosis, hyperfibrinolysis, and strong systemic inflammatory response [5]. Our results suggest that a heart rate of >100 beats/min, hemoglobin levels of <70 g/L, prothrombin time of >15 s, AKI, and higher APACHE II scores are independent risk factors for myocardial injury with high predictive value.

There are some limitations to this study. First, it was a single-center retrospective study, and the data collection time was long, so bias was inevitable. All of the patients were admitted to the same hospital. Unified testing facilities, diagnosis, and treatment schemes may reduce potential bias. Second, insufficient tissue perfusion caused by shock is an important reason for organ dysfunction and poor prognosis in most critically ill patients. Although the research group in this study was specific to patients with traumatic hemorrhagic shock, it lacks novelty in conclusion. Third, we measured cTNI concentrations in most patients only once daily, thereby possibly missing smaller troponin elevations. A number of indicators should be included in future studies, such as heart fatty acid binding protein and indicators on computed tomography coronary angiography. Furthermore, future research may provide a reference for identifying the cause of myocardial injury.

In conclusion, our study shows that the incidence rate of myocardial injury in traumatic hemorrhagic shock is 42.4%, and a heart rate of >100 beats/min, hemoglobin levels of <70 g/L, prothrombin time of >15 s, AKI, and higher APACHE II scores are independent risk factors for myocardial injury after traumatic hemorrhagic shock. The clinical manifestations of myocardial injury after traumatic hemorrhagic shock were insidious, and the prognosis was poor. Early detection and effective intervention for the above risk factors could reduce the occurrence of myocardial injury and improve poor prognosis. In the future, large-scale prospective studies are needed to further clarify the epidemiology, prevention, diagnosis, therapy, and prognosis of myocardial injury after traumatic hemorrhagic shock.

## Figures and Tables

**Table 1:** General and clinical characteristics of the patients with traumatic hemorrhagic shock.

Characteristics	*n* = 314
Male (*n*, %)	152 (48.4)
Age [M (P_25_–P_75_), years]	63.0 (49.8–82.0)
Preadmission conditions (*n*, %)	
Coronary heart disease	14 (4.5)
Hypertension	102 (32.5)
Diabetes mellitus	45 (14.3)
Cerebral hemorrhage	26 (8.3)
Gastrointestinal hemorrhage	5 (1.6)
Antiplatelet drugs	48 (15.3)
Anticoagulant drugs	7 (2.2)
Nonsteroidal anti-inflammatory drugs	20 (6.4)
Causes of trauma (*n*, %)	
Falling from height	47 (15.0)
Road traffic accident	95 (30.3)
Falling from a standing position	146 (46.5)
Others (crush, stab, animal bite)	26 (8.3)
Main bleeding site (*n*, %)	
Thoracic	55 (17.5)
Abdominal	34 (10.8)
Pelvic	31 (9.9)
Limbs	145 (46.2)
Others (blood vessels, skin, and soft tissue)	50 (15.9)
ISS [M (P_25_–P_75_)]	25.0 (16.0–34.0)
APACHE Ⅱ score [M (P_25_–P_75_)]	18.0 (14.0–23.0)
Acute myocardial injury (*n*, %)	133 (42.4)
Acute myocardial infarction (*n*, %)	2 (0.6)
Acute kidney injury (*n*, %)	88 (28.0)
In hospital mortality (*n*, %)	30 (9.6%)
Length of stay in hospital (days)	16.0 (10.0–24.0)
Length of stay in ICU (days)	11.0 (5.0–18.0)

ISS, injury severity scores; APACHE Ⅱ, acute physiology and chronic health evaluation II; ICU, intensive care unit; M (P_25_–P_75_), median (25th percentile, 75th percentile).

**Table 2:** Comparison of risk factors between traumatic hemorrhagic shock patients with and without myocardial injury.

Characters	Non-Myocardial Injury *n* = 181	Myocardial Injury *n* = 133	Test Value	*p* Value
Male (*n*, %)	78 (43.1)	74 (55.6)	4.831	0.028
Age [M (P_25_–P_75_), years]	64.0 (50.5–81.5)	62.0 (48.5–82.0)	0.211	0.833
Preadmission conditions (*n*, %)				
Coronary heart disease	10 (5.5)	4 (3.0)	1.141	0.286
Hypertension	57 (31.5)	45 (33.8)	0.192	0.661
Diabetes mellitus	27 (14.9)	18 (13.5)	0.119	0.730
Cerebral hemorrhage	17 (9.4)	9 (6.8)	0.696	0.404
Gastrointestinal hemorrhage	4 (2.2)	1 (0.8)	1.040	0.308
Antiplatelet drugs	29 (16.0)	19 (14.3)	0.178	0.673
Anticoagulant drugs	5 (2.8)	2 (1.5)	0.557	0.455
Nonsteroidal anti-inflammatory drugs	13 (7.2)	7 (5.3)	0.473	0.491
Causes of trauma (*n*, %)				
Falling from height	19 (10.5)	28 (21.1)	6.711	0.01
Road traffic accident	49 (27.1)	46 (34.6)	2.052	0.152
Falling from a standing position	99 (54.7)	47 (35.3)	0.028	0.867
Others (crush, stab, animal bite)	14 (7.7)	12 (9.0)	0.167	0.682
Main bleeding site (*n*, %)				
Thoracic	26 (14.4)	29 (21.8)	2.937	0.087
Abdominal	12 (6.6)	22 (16.5)	7.800	0.005
Pelvic	13 (7.2)	18 (13.5)	3.476	0.062
Limbs	105 (58.0)	40 (30.1)	24.073	<0.001
Others (blood vessels, skin, and soft tissue)	25 (13.8)	25 (18.8)	1.423	0.233
Mean arterial pressure [M (P_25_–P_75_), mmHg]	70.3 (63.3–80.8)	67.3 (53.3–77.2)	2.977	0.003
Heart rate [M (P_25_–P_75_), beats/minutes]	99.0 (84.8–114.0)	116.0 (102.5–130.5)	6.164	<0.001
Laboratory test				
Leukocyte count [M (P_25_–P_75_), ×10^9^/L]	9.7 (7.0–12.1)	10.2 (8.1–14.8)	2.195	0.028
Neutrophil count [M (P_25_–P_75_), ×10^9^/L]	8.3 (5.9–11.1)	9.3 (6.6–13.7)	1.567	0.117
Hemoglobin (Mean ± SD, g/L)	101.3 ± 19.2	90.9 ± 25.4	4.167	<0.001
Platelet count [M (P_25_–P_75_), ×10^9^/L]	159.5 (121.0–206.0)	135.0 (85.0–168.5)	3.840	<0.001
Serum creatinine [M (P_25_–P_75_), μmol/L]	65.5 (56.8–89.3)	80.0 (60.3–113.5)	3.223	0.001
Total bilirubin [M (P_25_–P_75_), μmol/L]	15.1 (11.6–21.1)	17.0 (8.6–23.0)	0.102	0.919
Prothrombin time [M (P_25_–P_75_), s]	13.8 (12.8–14.9)	14.8 (13.3–16.6)	6.561	<0.001
Fibrinogen [M (P_25_–P_75_), mg/dL]	249.0 (160.5–278.8)	154.0 (119.8–182.0)	5.572	<0.001
D-dimer [M (P_25_–P_75_), ng/mL]	3205.0 (1212.3–5354.8)	7462.0 (3294.0–16,961.3)	5.356	<0.001
pO_2_/FiO_2_ ratio [M (P_25_–P_75_), mmHg]	333.7 (255.6–387.5)	291.9 (207.2–362.4)	2.514	0.012
Serum lactate [M (P_25_–P_75_), mmol/L]	2.2 (1.7–3.3)	3.4 (1.9–6.7)	4.920	<0.001
Serum chloride [M (P_25_–P_75_), mmol/L]	106.7 (103.8–109.5)	111.8 (107.9–113.6)	3.192	0.001
Uric acid [M (P_25_–P_75_), μmol/L]	301.0 (186.8–453.0)	291.0(230.0–378.3)	2.563	0.01
C-reactive protein [M (P_25_–P_75_), mg/L]	47.0 (11.6–82.3)	57.8 (22.2–90.7)	0.280	0.779
Serum Procalcitonin [M (P_25_–P_75_), ng/mL]	0.7 (0.2–2.4)	3.3 (0.4–8.6)	2.245	0.025
cTNI [M (P_25_–P_75_), ng/mL]	0.007 (0.003–0.02)	0.4 (0.2–0.7)	12.411	<0.001
B-type Natriuretic Peptide [M (P_25_–P_75_), pg/mL]	47.0 (25.0–94.8)	68.0 (28.0–230.0)	0.932	0.351
LVEF [M (P_25_–P_75_), %]	66.4 (61.0–70.0)	64.0 (60.0–68.0)	1.642	0.101
Organ dysfunction (*n*, %)				
ARDS	40 (22.1)	49 (36.8)	8.204	0.004
AKI	33 (18.2)	55 (41.4)	20.319	<0.001
Acute liver injury	29 (16.0)	54 (40.6)	23.818	<0.001
ISS [M (P_25_–P_75_)]	14.0 (14.0–25.0)	22.0 (14.0–34.0)	4.780	<0.001
APACHE Ⅱ score [M (P_25_–P_75_)]	16.0 (12.0–19.0)	19.0 (16.0–23.0)	5.758	<0.001

cTNI, cardiac troponin I; LVEF, Left ventricular ejection fraction; ARDS, acute respiratory distress syndrome; AKI, acute kidney injury; ISS, injury severity scores; APACHE Ⅱ, acute physiology and chronic health evaluation II; M (P_25_–P_75_), median (25th percentile, 75th percentile).

**Table 3:** Univariate and multivariate logistic regression analysis on risk factors of myocardial injury after traumatic hemorrhagic shock.

	Univariate Analysis		Multivariate Analysis	
Characters	OR (95% CI)	*p*	OR (95% CI)	*p*
Male	1.66 (1.06–2.60)	0.028		
Falling from height	2.27 (1.21–4.28)	0.011		
Bleeding of abdominal	2.79 (1.33–5.87)	0.007		
Bleeding of limbs	0.31 (0.19–0.50)	<0.001		
Heart rate > 100 beats/min	4.35 (2.59–7.30)	<0.001	3.33 (1.56–7.09)	0.002
Mean arterial pressure	0.98 (0.97–0.99)	0.006		
Leukocyte count	1.05 (1.00–1.09)	0.026		
Hemoglobin < 70 g/L	5.74 (2.63–12.54)	<0.001	3.50 (1.15–10.6)	0.027
Platelet count	0.995 (0.991–0.998)	0.001		
Prothrombin time > 15 s	4.61 (2.61–8.13)	<0.001	2.39 (1.12–5.10)	0.024
Fibrinogen	0.995 (0.993–0.997)	<0.001		
D-dimer	1.00 (1.00–1.00)	<0.001		
Total bilirubin	0.99 (0.98–1.02)	0.823		
Uric acid	1.002 (1.001–1.004)	0.013		
Serum chloride	1.07 (1.02–1.12)	0.003		
pO_2_/FiO_2_ ratio	0.99 (0.98–0.99)	0.009		
Serum lactate	1.28 (1.14–1.45)	<0.001		
Serum Procalcitonin	1.06 (0.97–1.16	0.184		
ARDS	2.06 (1.25–3.38)	0.005		
AKI	3.16 (1.90–5.27)	<0.001	2.75 (1.27–5.93)	0.010
Acute liver injury	3.58 (2.12–6.07)	<0.001		
ISS	1.06 (1.03–1.08)	<0.001		
APACHE II score	1.14 (1.09–1.19)	<0.001	1.08 (1.01–1.15)	0.018

ARDS, acute respiratory distress syndrome; AKI, acute kidney injury; ISS, injury severity score; APACHE Ⅱ, acute physiology and chronic health evaluation II.

## Data Availability

The datasets generated and/or analyzed during the current study are not publicly available but are available from the corresponding author on reasonable request.
